# Identification of differentially expressed genes through RNA sequencing in goats (*Capra hircus*) at different postnatal stages

**DOI:** 10.1371/journal.pone.0182602

**Published:** 2017-08-11

**Authors:** Yaqiu Lin, Jiangjiang Zhu, Yong Wang, Qian Li, Sen Lin

**Affiliations:** Key Laboratory of Sichuan Province for Qinghai-Tibetan Plateau Animal Genetic Reservation and Exploitation, Chengdu, Sichuan, P. R. China; Kunming University of Science and Technology, CHINA

## Abstract

Intramuscular fat (IMF) content and fatty acid composition of longissimus dorsi muscle (LM) change with growth, which partially determines the flavor and nutritional value of goat (*Capra hircus*) meat. However, unlike cattle, little information is available on the transcriptome-wide changes during different postnatal stages in small ruminants, especially goats. In this study, the sequencing reads of goat LM tissues collected from kid, youth, and adult period were mapped to the goat genome. Results showed that out of total 24 689 Unigenes, 20 435 Unigenes were annotated. Based on expected number of fragments per kilobase of transcript sequence per million base pairs sequenced (FPKM), 111 annotated differentially expressed genes (DEGs) were identified among different postnatal stages, which were subsequently assigned to 16 possible expression patterns by series-cluster analysis. Functional classification by Gene Ontology (GO) analysis was used for selecting the genes showing highest expression related to lipid metabolism. Finally, we identified the node genes for lipid metabolism regulation using co-expression analysis. In conclusion, these data may uncover candidate genes having functional roles in regulation of goat muscle development and lipid metabolism during the various growth stages in goats.

## Introduction

Intramuscular fat (IMF), the flecks and streaks of fat within the lean sections of meat, also known as marbling, is positively associated with juiciness and flavor. Optimal IMF content and fatty acid composition are also beneficial for human health. Genetic improvement of IMF deposition and fatty acid compositions are major goals of goat breeding. Comparing with research in cattle [1, 2], pigs [3, 4], chicken [5], and sheep [6–8], although peroxisome proliferator-activated receptor gamma (*PPARγ*) is related with IMF metabolism in goat (*Capra hircus*) mammary gland [9], few studies have focused on the molecular mechanisms of lipid metabolism in goat muscle.

A Number of genes are important for lipid metabolism in muscle. In pigs, peroxisome proliferator-activated receptor gamma coactivator-1α (*PGC1α*) is associated with IMF content [10]. HDL-binding protein (*HDLBP*) is also associated with IMF percentage [11]. In male Kazak sheep, the mRNA expression level of heart-type fatty acid binding protein (*FABP3*) is highly and positively correlated with IMF content, but that of *PPARγ* is highly and negatively correlated with IMF content [6]. In the bulls and steers, castration results in improved marbling of longissimus dorsi muscle (LM) by increasing the expression of lipid uptake genes *viz*. lipoprotein lipase (*LPL*), fatty acid translocase (*CD36*) and fatty acid transport protein 1 (*FATP1*), enhancing lipogenic genes *viz*. fatty acid synthase (*FASN*) and acetyl-CoA carboxylase α (*ACCα*), and down-regulating lipolytic genes *viz*. triglyceride lipase (*ATGL*) and monoglyceride lipase (*MGLL*) [12]. Obviously individual gene cannot illuminate the mechanism of regulation of IMF deposition, and studies employing transcriptome-wide mining of the functional genes in lipid metabolism are needed.

Moreover, no information is available on the alterations of the transcriptome vis-a-vis IMF content, particularly in goats. Considering that IMF content increased continuously with growth in sheep [6], it is hypothesized that the genes that are associated with lipid metabolism and that are expressed differentially during different postnatal stages in goat LM may provide us candidates for the regulation of IMF deposition beyond the development of muscle mass. In the present study, RNA sequencing was used for expression profiling of muscle tissue from 3 postnatal stages of Jianzhou Big-Eared goats. The results determined the differentially expressed genes (DEGs), and revealed potential candidates with important role in regulation of lipid metabolism.

## Material and methods

### Ethics statement

The animal care and use were performed according to the regulations for the Administration of Affairs Concerning Experimental Animals (Ministry of Science and Technology, China, revised in June 2004) and approved by the Institutional Animal Care and Use Committee, Southwest University for Nationalities, Chengdu, Sichuan, China.

### Animals, sampling, and RNA collection

The Jianzhou Big-Eared goats used in this experiment were obtained from the Jianyang Dageda animal husbandry Co. Ltd in Jianyang County, Sichuan, China. Methods for tissue collection have been described previously [13]. Briefly, nine healthy Jianzhou Big-Eared goats, three per stage, in the period of kid (2-month age, group G), youth (9-month age, group Y) and adult (24-month age, group C) were selected randomly. All the goats were fed freely in the same house with alfalfa, corn straw silage and supplemented with concentrate; the concentrate comprised of corn, soybean, wheat bran, rapeseed meal, and a mineral-vitamin mixture. The kids were nursed freely by the lactating goats. All the goats performed well during experiments. The LM tissues were collected by a veterinarian after slaughter. All the tissue samples were harvested within 20 min of slaughter under sterile conditions and immediately frozen in liquid nitrogen until RNA extraction.

IMF was determined by Soxhlet method using anhydrous ether as the solvent, and expressed as percentage of dry weight. Extraction of total RNA, purification of mRNA, and construction of cDNA library were performed by Novogene (Beijing, China). In brief, total RNA was obtained from LM using the Trizol method and treated with RNase-free DNase. The quality and purity of total RNA were analyzed by agarose gel electrophoresis, Bioanalyzer 2100 and Nanodrop system (Agilent, CA, USA). The RNA Integrity Number (RIN) of RNA were 8.4, 8.1, 8.0 from kids; 7.7, 8.1, 7.8 from youth; and 7.2, 7.7, 8.2 from adults; respectively. Qubit^®^ 2.0 fluorometer was used for determination of RNA concentration.

### cDNA library construction and Illumina sequencing

Total RNA obtained from goats of the same group was pooled in equal quantities (1 μg mRNA per goat). mRNA was isolated with poly-T-oligo-attached magnetic beads (Invitrogen, USA). Following purification, the mRNA was fragmented using fragmentation buffer. Random hexamers were then used for first cDNA strand synthesis, following which the second strand of cDNA was synthesized after the supplementation of buffer, dNTPs, and DNA Polymerase I. The cleaved cDNA purified by AMPure XP beads (Illumina, San Diego, USA) was modified by end-repair, and addition of A-tail and adapter. PCR enrichment was performed following the size selection of fragments to create the final cDNA library. The cDNA library was then quantified by Qubit 2.0 fluorometer and diluted to a terminal concentration of 1 ng/μl. Agilent 2100 Bioanalyzer system was then used for identification of insert size following quantification of concentration by quantitation real-time PCR (qPCR). Pair-end sequencing of 2×150 bp was then performed on an Illumina HiSeq 4000 equipment following the vendor’s recommended protocol for 1 lane.

### Data filtering and sequence assembly

Prior to assembly, the raw reads (raw data) were determined by fastqc [14] and filtered by removing the adapter sequences and potential contamination, i.e. reads with unknown base (N) greater than 10% and low quality sequences (<Q20) [15], to obtain clean data. All of the downstream analyses were based on high quality clean data. TopHat2 method was used for sequence mapping and assembly with the complete goat genome (CHIR_1.0) as the reference sequence (ftp://ftp.ncbi.nlm.nih.gov/genomes/Capra_hircus/) [16].

### Gene annotation and determination of expression level

Assembled Unigenes were annotated to the reference goat genome using BLAST. Unigene expression level was recorded according to the sum frequencies of compared reads. Expected number of fragments per kilobase of transcript sequence per million base pairs sequenced (FPKM) was used for expression normalization [17]. The goat genome was aligned to the Gene Ontology (GO, http://www.geneontology.org/) of other species, including human, bovine, mice et al., to build the GO database available for goat which was then used for GO analysis in the present study [18].

### Analysis of differentially expressed genes among various growth stages

DEGs were analyzed using DESeq method following the normalization of read count by Trimmed Mean of M values (TMM) [19]. DEGs were selected according to |log2(fold-change)| > 1 and *q* value < 0.005 (normalized *p* value), which were then used for GO analysis. The software of Short Time-series Expression Miner (STEM) was used for expression pattern analysis by the “Normalize data” method; the “maximum number of model profiles” was set at 20 and the “maximum unit change in model profiles between time points” was set at 2. The significance level was set at 0.05, and the minimum correlation with the clustering profiles was set at 0.7 [20]. Gene co-expression analysis [21, 22] was performed to track the interactions among the DEGs profiles. Pearson correlation was performed for each pair of genes and the significantly correlated pairs were used to construct a network with a threshold of 0.92 [23] using a Perl script. The number of correlated genes was used for selection of the node genes.

### Quantitative real-time PCR

cDNA was synthesized from 0.5 μg of total RNA using the PrimeScript^™^ RT kit with gDNA Eraser (Takara, Japan) which removes genomic DNA contamination, according to the manufacturer’s instructions. qPCR was performed according to manufacturer’s instructions using SYBR green (SYBR^®^ Premix Ex Taq^™^ II, Perfect Real Time, Takara, Japan). The sequences of the primers are shown in the S1 Table. Peptidylprolyl isomerase B (*PPIB*) was chosen as the internal control gene, and amplified using the primer sequences reported previously [13]. Three independent biological replicates were analyzed per sample by qPCR. The relative gene expression was calculated using the 2^-ΔΔCt^ method. Significance for RNA expression between different ages was determined by one-way ANOVA using SPSS22.0. Significance was declared at *P*<0.05. Pearson correlation was performed for each pair of genes.

### GEO accession numbers

The data obtained from RNA sequencing studies were deposited in the Gene Expression Omnibus database (http://www.ncbi.nlm.nih.gov/geo/) and Sequence Read Archive (https://www.ncbi.nlm.nih.gov/sra) at NCBI. The accession number for GEO and SRA are GSE85014 and SRP068688.

## Results

### Intramuscular fat determination

**Fig 1:**
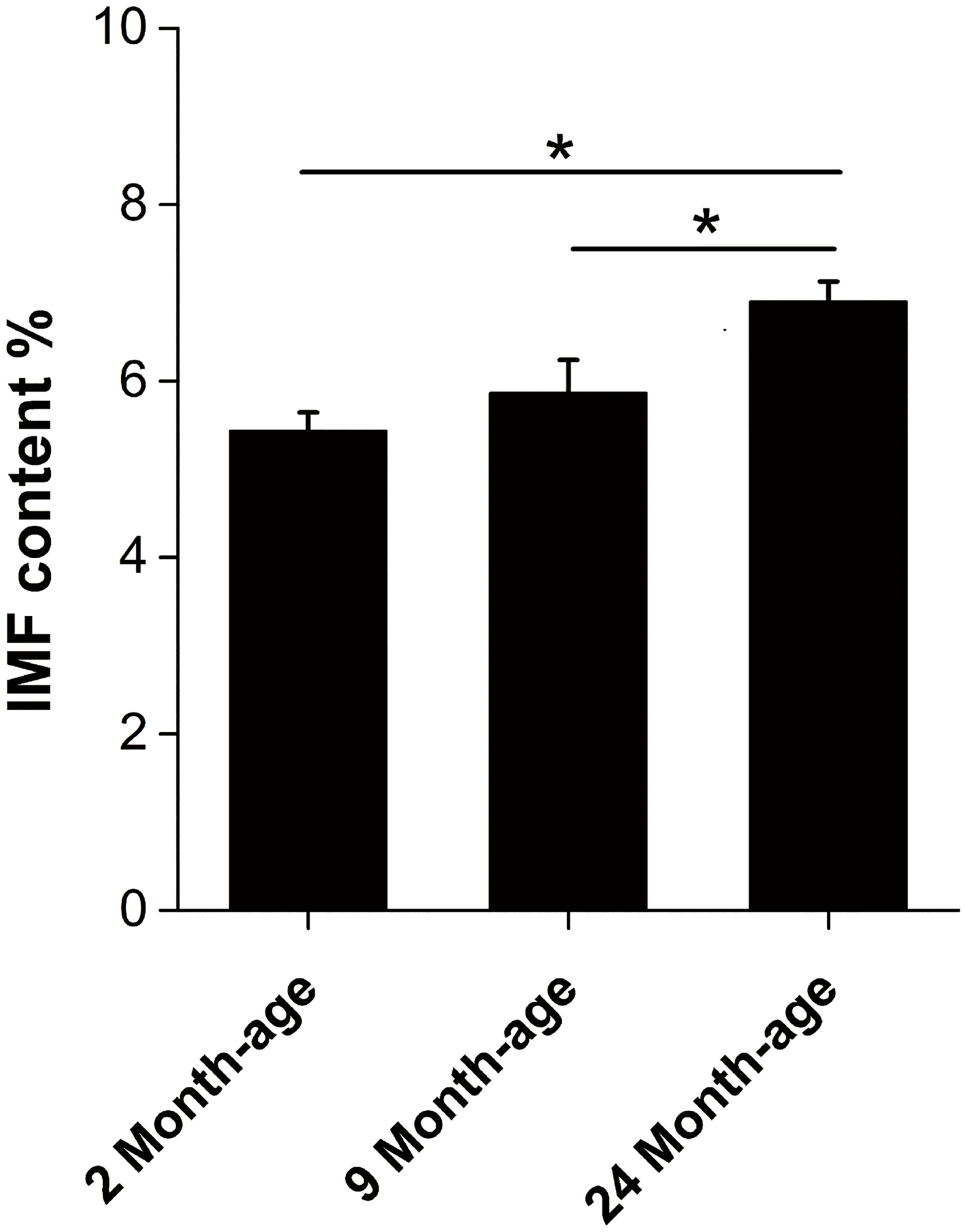
IMF content in 2-month age, 9-month age and 24-month age goats. **IMF, intermuscular fat**. * represents *P*<0.05.

The IMF content increased continuously with the development of goat muscle and peaked at 24-month age, which was significantly higher than that at 9-month age and 2-month age. The IMF content at 9-month age was higher than that at 2-month age, although not statistically significant (Fig 1).

### Illumina sequencing and assembly

Illumina sequencing of goat muscle tissue yielded a total of 47 590 558, 44 719 748, 55 361 278 clean reads with 100% valid data in the groups “G”, “Y” and “C”, containing 7.14 G, 6.71 G, and 8.3 G clean bases, which mapped to 73.444%, 71.84%, and 70.68% of the reference goat genome sequence, respectively.

In the samples from group G, 84.1%, 5.8%, and 10.2% of the total reads were mapped to exon, intergenic and intron regions, respectively. The samples from the groups “Y” and “C” covered 85.2%, 5.6%, 9.2% and 85.7%, 5.5%, 8.8%, respectively.

Ten chromosomes, including 1, 2, 3, 4, 5, 6, 7, 8, 11 and X, were covered by the total clean reads of the samples across the three stages. While chromosome 1, 2 and X had the most of clean reads, chromosomes 7 and 11 had the least clean reads.

### Analysis of genes related to lipid transport and metabolism with high expression levels

**Fig 2:**
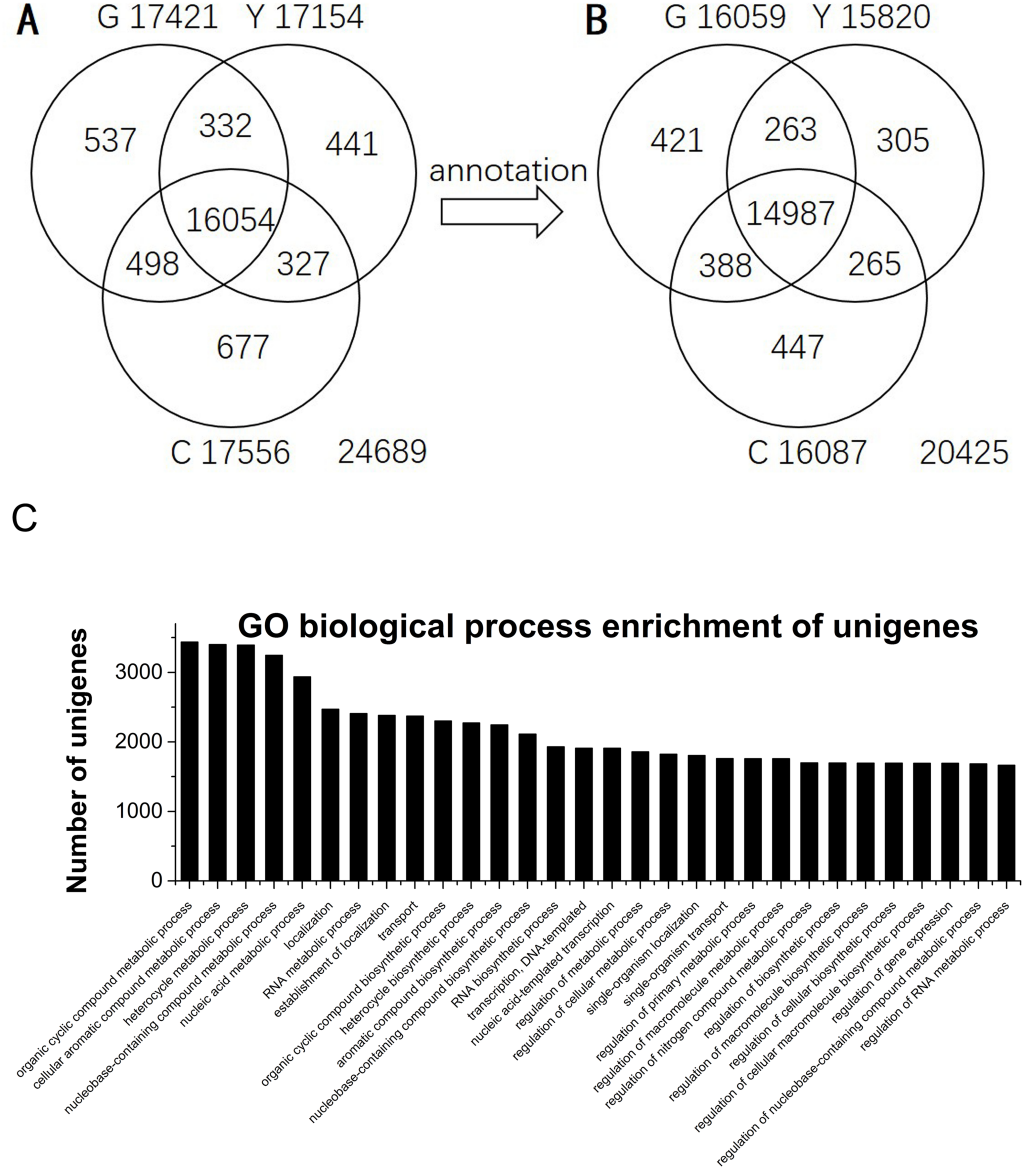
Unigenes distribution in different periods of goat longissimus dorsi muscle at different postnatal stages. A. Unigenes distribution in kid (G), young (Y) and adult (C) goats muscle. B. The distribution of annotated Unigenes in goat muscle at different postnatal stages. C. Top 30 GO biological process categories by GO enrichment of defined Unigenes. Significance level was *P*<0.05.

Illumina sequencing uncovered 24 689 Unigenes, including 17 421 Unigenes in the samples from group G, 17 154 Unigenes in the samples from group Y and 17 556 Unigenes in the samples from group C (Fig 2A). Samples from the group G expressed 1 086 Unigenes with FPKM greater than 60, and had 2 797 Unigenes with FPKM between 15 and 60, accounting for 4.4 and 11.33% of the total Unigenes, respectively. However, most of the Unigenes (20 806) in the group G were expressed below 15 FPKM (accounting for 84.27% of the total Unigenes). According to the same FPKM classification the samples from the groups Y and C expressed 1 065, 2 701, 20 923 Unigenes (accounting for 4.31%, 10.94% and 84.75%) and 1 104, 2 614, 20 917 Unigenes (accounted for 4.47%, 10.59% and 84.94%), respectively (S1 File).

Of 20 425 defined Unigenes, 16 059, 15 820 and 16 087 Unigenes, and 421, 305 and 447 uniquely expressed defined genes were identified in the samples of group G, Y and C, respectively (Fig 2B). These Unigenes were enriched into 199 GO categories, of which the organic cyclic compound metabolic processes contained most of the Unigenes among 89 biological processes (Fig 2C). (S1 File).

16002 defined Unigenes were annotated to three main GO categories, including biological process, cellular component and molecular function, of which 619 defined genes were associated with lipid metabolic process (S1 File). Ribosomal protein S3A (*RPS3A*), nebulin, transcript variant X1 (*NEB*), and fructose-1,6-bisphosphatase 2 (*FBP2*) were the top three genes in the group G and C samples with FPKM of 791.268, 697.603, 404.234 and 576.974, 599.270 and 515.530, respectively. Although *RPS3A* and *NEB* were also the top two genes (FPKM of 687.634 and 606.082, respectively) in the samples of group Y, myosin light chain kinase 2, transcript variant X1 (*MYLK2*) was the third highest expressed gene (FPKM = 472.406), closely followed by *FBP2* with FPKM of 360.732.

Within the 79 defined genes associated with the fatty acid metabolic process GO, enoyl-CoA hydratase (trifunctional protein), beta subunit (*HADHB*), vimentin (*VIM*) and acetyl-CoA acetyltransferase 1 (*ACAT1*) had the highest expression in the samples of groups G and Y with FPKM of 290.692, 247.270, 236.088, and 194.394, 203.159, 252.401, respectively. In the group C, the top three genes were *ACAT1*, *HADHB* and *HADHA*, with FPKM of 260.667, 253.297 and 180.980, respectively. (S1 File).

Within the fatty acid biosynthetic process GO, the top three genes were *HADHB*, vimentin and *ACAT1* in all the stages, with FPKM of 290.692, 247.270 and 236.087 in group G samples; 194.393, 203.159, and 252.401 in group Y samples; and 253.297, 158.729, and 260.667 in group C samples, respectively. (S1 File).

### Identification of differential gene expression patterns in goat muscle

Comparing with the group G samples, 25 uniquely defined DEGs were identified in the group Y samples, including 13 upregulated and 12 down-regulated DEGs. Myosin, heavy chain 13, skeletal muscle (*MYH13*), interferon-induced protein with tetratricopeptide repeats 1 (*IFIT1*), methyltransferase like 21C (*METTL21C*) and serpin peptidase inhibitor, clade E (nexin, plasminogen activator inhibitor type 1), member 1, transcript variant X1 (*SERPINE1*), early growth response 1 (*EGR1*), estrogen-related receptor gamma, transcript variant X3 (*ESRRG*) were the most three fold-change-modified genes, with the fold-changes of 95.339, 3.929, 3.869 and 0.213, 0.223, 0.295, respectively (S2 Table, S1 File).

28 upregulated genes were identified in the group C samples when compared with the group G samples, the top three genes being 40S ribosomal protein S25-like (*RPS25*), *MYH13* and cartilage oligomeric matrix protein (*COMP*) with fold-changes of 148.673, 93.183 and 14.611, respectively. According to the same fold-change method, the most three fold-change-modified genes among the 36 down-regulated genes were transcription factor BTF3-like (*LOC102186300*), *SERPINE1* and nuclear receptor subfamily 4, group A, member 2 (*NR4A2*) with fold-changes of 0.057, 0.100 and 0.165, respectively (S3 Table, S1 File).

There were 37 up-regulated genes and 36 downregulated genes in the group C samples, when compared with the group Y samples, of which the most three fold-change-modified genes according to the fold-change were *RPS25* (88.279), *COMP* (12.415), prostate androgen-regulated mucin-like protein 1 (*PARM1*) (7.454) and BOLA class I histocompatibility antigen, alpha chain BL3-7-like (LOC102177715) (0.024), AT rich interactive domain 5B (MRF1-like), transcript variant X1 (*ARID5B*) (0.190), 6-phosphofructo-2-kinase/fructose-2,6-biphosphatase 3 (*PFKFB3*) (0.204), respectively (S4 Table, S1 File).

**Fig 3:**
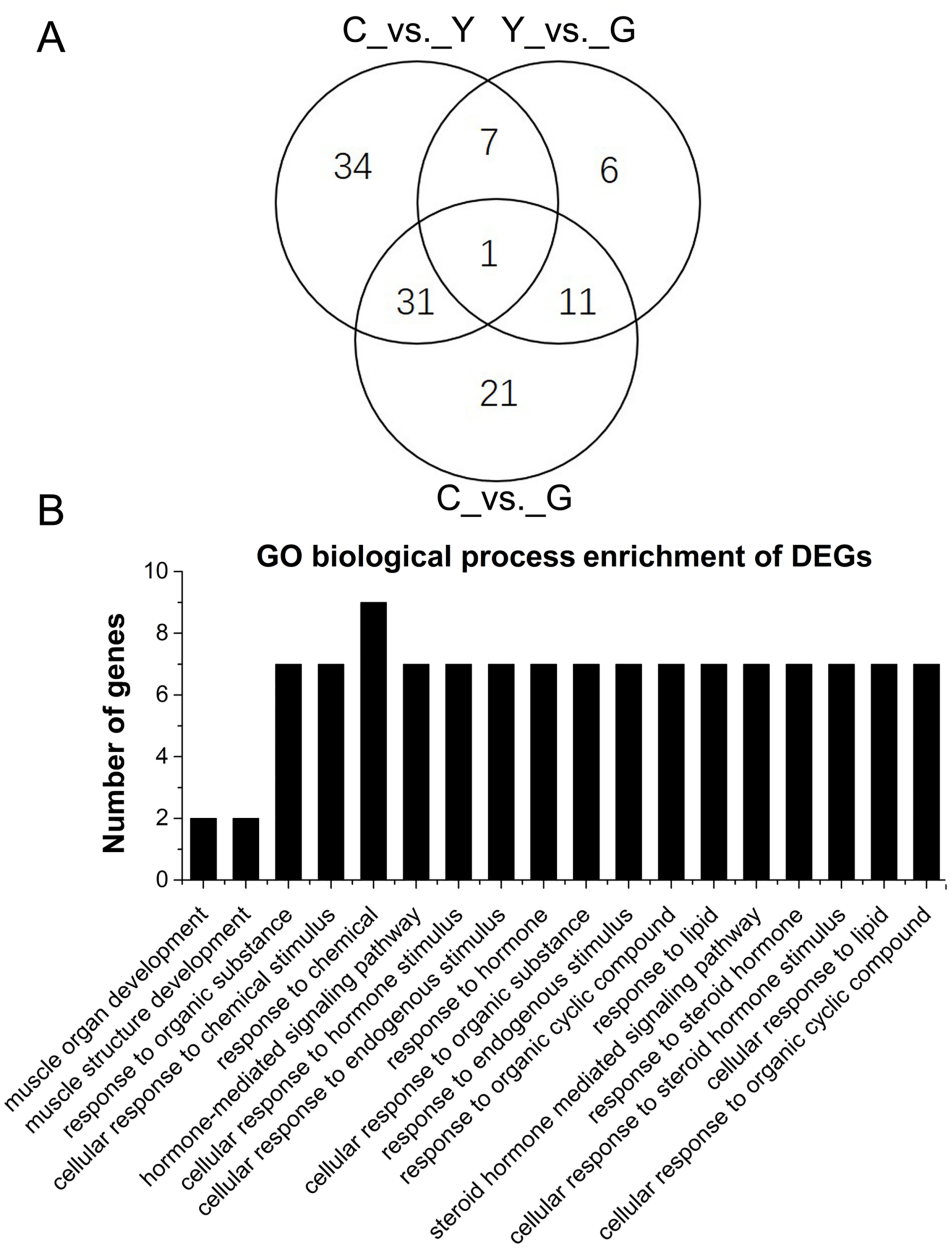
Distribution of differentially expressed genes (DEGs) of goat longissimus dorsi muscle at different postnatal stages. A. Distribution of differentially expressed genes. C_vs._G, the differentially expressed genes in the adult samples (C) compared with the kid (G) samples. C_vs._Y, the DEGs in the “C” samples compared with the youth (Y) samples. Y_vs._G, the DEGs in the group Y samples compared with the group G samples. B. The GO biological process enrichment of defined Unigenes. Significance level was *P*<0.05.

There were 12 DEGs shared between “C_vs._G” (the DEGs in the group C samples compared with the group G samples) and “Y_vs._G”. 52 and 13 DEGs were uniquely identified in “C_vs._G” and “Y_vs._G”, respectively. According to similar classification, the number of genes were 8, 65 and 17 for “C_vs._Y” and “Y_vs._G”, and 32, 32, 41 for “C_vs._G” and “C_vs_Y”, respectively. Only one gene, *NR4A3*, was shared by all the groups. 34, 6 and 21 genes were uniquely differentially expressed in “C_vs._Y”, “Y_vs._G” and “C_vs._G”, respectively (Fig 3A).

Four genes were associated with lipid metabolic process GO, including microtubule associated monooxygenase, calponin and LIM domain containing 2, transcript variant X1 (*MICAL2*), nuclear receptor subfamily 1, group D, member 2 (*NR1D2*), glycerol-3-phosphate dehydrogenase 2 (mitochondrial) (*GPD2*), also related to fatty acid metabolic process GO, and salt-inducible kinase 1 (*SIK1*), with FPKM of 5.259, 31.569, 41.547, 42.225 in the group G samples, 3.061, 43.653, 41.829, 30.487 in the group Y samples and 14.366, 19.475, 13.944, 10.430 in the group C samples, respectively. However, no genes were related to the fatty acid biosynthetic process GO. Among the samples from the three stages, a total of 117 DEGs, including 111 defined genes, were identified, that enriched into 19 GO categories, including 18 biological processes and 1 molecular function, of which the DEGs associated with response to chemicals contained most of genes (Fig 3B).

**Fig 4:**
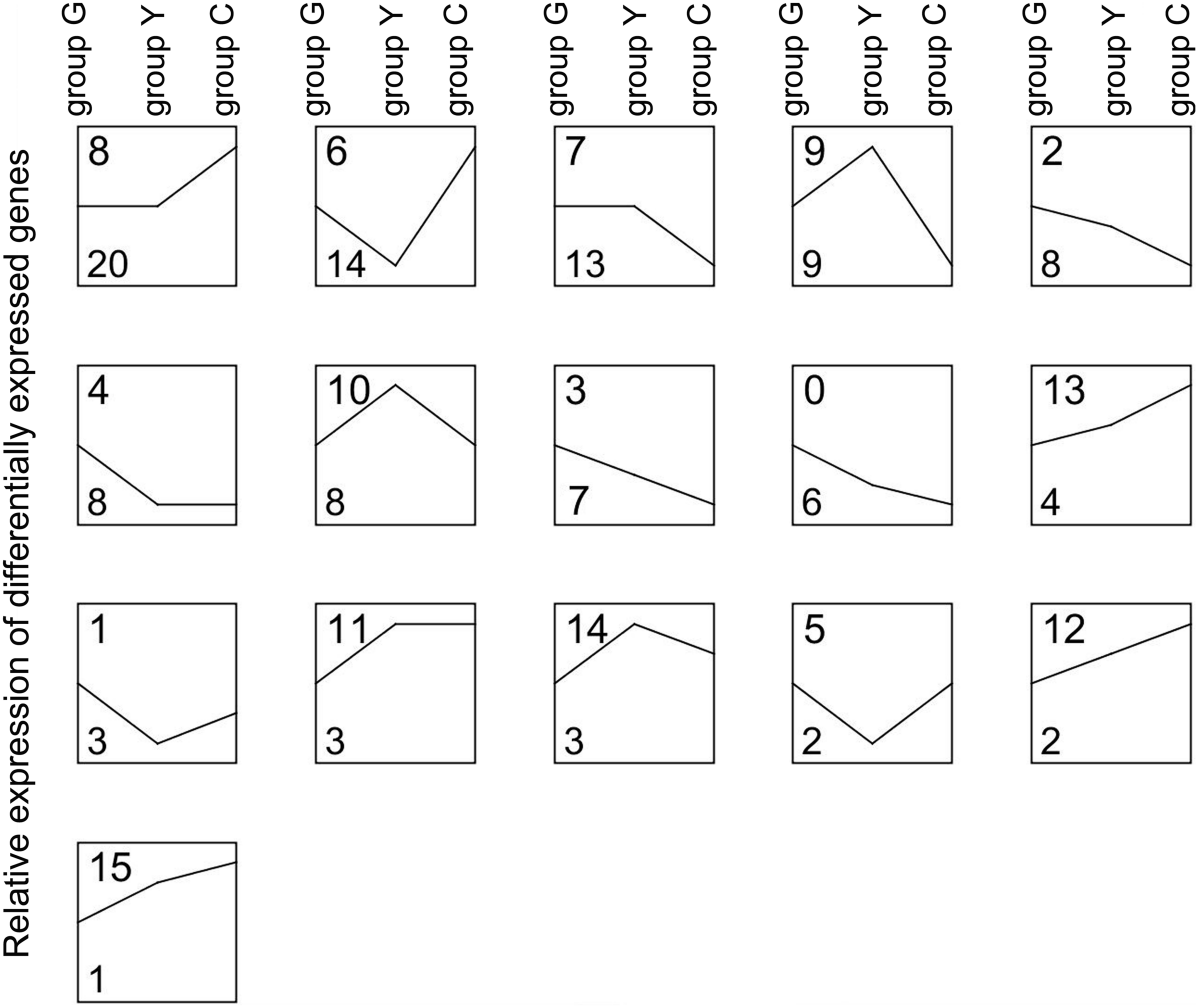
Series-cluster analysis of differentially expressed genes (DEGs) during growth in goats at different postnatal stages using STEM software. The significant level was set at 0.05 and the minimum correlation at 0.7; the “maximum number of model profiles” was set at 20 and the “maximum unit change in model profiles between time points” was set at 2. group G, kid goats; group Y, young goats; group C, adult goats. The number in the top left-hand corner of a profile box is the pattern ID number. The number in the lower left corner of the profile box is the number of genes.

According to the expression level, the 111 defined DEGs were significantly correlated with 16 possible expression clustering patterns using the software of STEM. 21 genes transcripts (patterns 0, 2 and 3) decreased continuously with the growth of goats, whereas 7 gene transcripts (patterns 12, 13 and 15) were continuously increased. 33 genes were stable in group Y, of which 20 genes were turned up (pattern 8) and 13 genes were turned down (pattern 7) in the group C samples. After changing in the period corresponding to group Y, 11 genes (patterns 4 and 11) maintained a similar expression level in the period corresponding to group C. While 19 genes were increased in the period corresponding to group C period after a decrease in the period corresponding to group Y period (patterns 1, 5 and 6), the converse expression trend contained 20 genes (patterns 9, 10 and 14). A total of 47 DEGs were significantly upregulated in the adult stage (patterns 6, 8, 11, 12, 13, 14 and 15), whereas 54 DEGs were significantly decreased in this stage (patterns 0, 1, 2, 3, 4, 7 and 9). Ten DEGs maintained the stable expression level between the periods corresponding to groups C and G despite the significant change in expression during the period corresponding to group Y (patterns 5 and 10) (Fig 4, S1 File).

**Fig 5:**
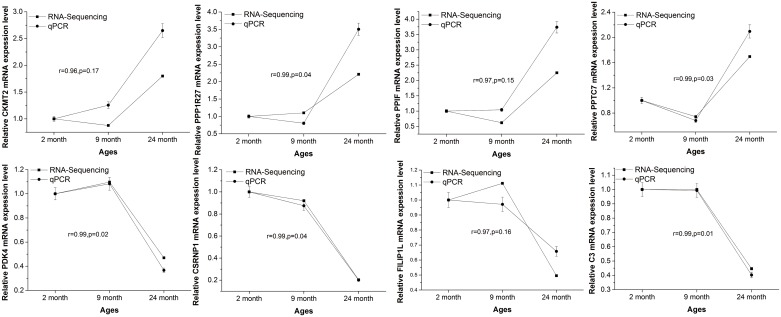
Quantitative real-time PCR (qPCR) validation of differentially expressed genes from RNA sequencing. *C3*, complement component 3; *CKMT2*, creatine kinase, mitochondrial 2 (sarcomeric); *FILIP1L*, filamin A interacting protein 1 like, transcript variant X1; *PPIF*, peptidylprolyl isomerase F; *PPP1R27*, protein phosphatase 1, regulatory subunit 27; *PPTC7*, PTC7 protein phosphatase homolog (S. cerevisiae); *PDK4*, pyruvate dehydrogenase kinase, isozyme 4; *CSRNP1*, cysteine-serine-rich nuclear protein 1, transcript variant X2. *PPIB*, peptidylprolyl isomerase B was chosen as the internal control genes. Pearson correlation was performed for each pair of genes.

Co-expression analysis revealed that creatine kinase, mitochondrial 2 (sarcomeric) (*CKMT2*), pyruvate dehydrogenase kinase, isozyme 4 (*PDK4*), and prostate androgen-regulated mucin-like protein 1 (*PARM1*) were the top three genes correlating with most number of DEGs (54, 54, and 53 genes, respectively) (S5 Table, S1 File). In sorder to identify the quality of RNA sequencing, we confirmed the mRNA expression of complement component 3 (*C3*), *CKMT2*, filamin A interacting protein 1 like, transcript variant X1 (*FILIP1L*), peptidylprolyl isomerase F (*PPIF*), protein phosphatase 1, regulatory subunit 27 (*PPP1R27*), *PTC7* protein phosphatase homolog (*S*. *cerevisiae*) (*PPTC7*), *PDK4* and cysteine-serine-rich nuclear protein 1, transcript variant X2 (*CSRNP1*) by qPCR. Similarities in the expression patterns were observed in the results by qPCR and RNA sequencing. Strong correlations were identified for all the 8 genes (r>0.90), although not statistically significant in *CKMT2* (*P* = 0.17), *PPIF* (*P* = 0.15) and *FILIP1L* (*P* = 0.16) (*P* > 0.05) (Fig 5).

## Discussion

Next-generation sequencing has facilitated the research on the regulation of IMF deposition in chicken [24], cattle [25], and pigs [26]. Despite the characterization of the complete transcriptome of the goat mammary gland [27] and skin [28], to date, no information is available on the transcriptome for goat muscle development. Based on the goat genome sequence [16], in the present study we found that IMF deposition was positively correlated with growth of goats, consistent with the data in sheep [6]. The highest IMF content was observed in adult goats, and was significant higher than that in kids and in young goats. It implies that the DEGs, also genes encoding proteins between 3 month and 24 month, and 9 month and 24 month, could be associated with IMF deposition in goats. In the present study, 32 DEGs were shared by “C_vs._G” and “Y_vs._C”, which may prove candidate genes for the regulation of IMF deposition during growth in goats. We also realized that the same 3 goats across different stages using biopsy method instead of slaughter may result in more solid DEGs collection in the future.

We also recognized that the development of muscle mass, rather than IMF deposition, could be the main factor affecting the transcriptome patterns associated with goat development. However, by GO classification following STEM and co-expression analyses, we obtained the genes associated with lipid metabolism, predicted to be mainly responsible for IMF deposition in goat muscle. On the other hand, although we had tried our best to exclude the effects of diet, the discrepancies in diet at different ages in goats may be another contributor to differential gene expression. In the present study, the goats were fed freely upon alfalfa, corn straw silage and concentrate. Identical management practices are beneficial for reducing the effects induced by the differences in diet. Although great number of such controlled in vivo experiments is needed in the future for identifying the exact reasons for the differential expression of genes, our suggests candidate genes that may be crucial for growth and lipid metabolism in goats.

Dong et al. (2013) were the first to publish genomic information on goats. Compared with the de novo sequencing method [27, 29], the use of goat genome as reference sequences would have, undoubtedly, resulted in a better assembly and annotation. In the present study, we mapped the data of transcriptome sequence to goat genome, and identified a total of 24 689 Unigenes supporting 20 425 annotated genes (82.73%). This method allows more exactitude in the results of analysis of differential gene expression.

Although 111 defined genes were identified to be expressed differentially during growth in goats, only 4 genes were associated with lipid metabolism. The activation of SIK1 suppresses metastasis through the AMP-activated protein kinase (AMPK) pathway [30], and inhibits lipid synthesis in human hepatoma cells [31]. Considering the continuous decrease of *SIK1* during muscle development, it is predicted that *SIK1* may be involved in lipid metabolism in muscle via the control of the AMPK pathway. However, more research for finding an exact mechanism underlying the expression pattern is needed.

*NR4A3* is the only gene showing significant differential expression during growth in goats. As a member of the nuclear orphan receptors *NR4A*-family, it participates in cell cycle, apoptosis, inflammation, atherogenesis, metabolism, DNA repair and tumorigenesis [32]. In humans, elevation in *NR4A3* reduces the expression of insulin gene [33], which is closely associated with lipid metabolism. The EWSR1 (EWS RNA binding protein 1)/NR4A3 fusion protein of extraskeletal myxoid chondrosarcoma activates PPARγ [34] which is crucial for lipid metabolism regulation [35, 36]. Thus, it is hypothesized that the continuous increase in *NR4A3* expression with growth in goats may be correlated with IMF deposition. Obviously, more controlled molecular experiments, such as RNA silencing and over-expression studies, will be beneficial in addressing the precise role of *NR4A3* in regulating lipid deposition in adipocytes.

Although 111 defined genes undergoing differential expression were identified, the key genes related to lipid metabolism during muscle development remains unclear. Using series-cluster analysis, we identified 16 possible profiles representing the overall expression patterns, and also classified the genes related to lipid metabolism, including sterol regulatory element binding transcription factor 1 (*SREBP1*) (pattern 6), *FABP3* (pattern 6), adipocyte fatty acid-binding protein (*FABP4*) (pattern 4), *PGC1α* (pattern 8), forkhead box O1 (*FOXO1*) (pattern 7), Kruppel-like factor (*KLF4*) (pattern 0), *KLF10* (pattern 9) and so on. Previously, a close correlation between *SREBP1* and *FABP3* [37] was observed during IMF deposition. PGC1α can interact with FOXO1 to involve lipid metabolism in rats [38]; unexpectedly, in the present study converse expression pattern was identified between *PGC1α* and *FOXO1*. Although the differences between goats and rats may contribute to such divergence, evidence on direct interactions between PGC1α and FOXO1 in goat adipocytes may be beneficial for understanding their expression patterns in the future.

The use of co-expression analysis has been reported to be a suitable method for selection of regulatory gene [22]. The node genes correlating with the greatest number of genes were considered to be crucial for the regulation of lipid metabolism during growth in goats. Our analyses revealed that *PDK4* may regulate lipid metabolism during growth. As the last member of PDK family, *PDK4* is induced by increased lipid delivery [39], and is negatively correlated with the sensitivity of pyruvate dehydrogenase complex (*PDHC*) to pyruvate, a substrate for lipid metabolism, in skeletal muscle [40]. *PDK4* is also a negative regulator of glucose oxidation, and is involved in fatness [39]. Increased *PGC1α* enhances the expression of *PDK4* gene in mice [41]. These studies indicate that *PDK4* is important in lipid synthesis. However, unlike in mice, in the present study, after stable expression at youth stage, *PDK4* was significantly decreased at adult stage (pattern 7) despite the increase in *PGC1α* expression. Although the exact regulation mechanism between *PGC1α* and *PDK4* remains unclear, *PDK4* is predicted to be a novel crucial regulator of lipid metabolism during growth in goats.

We also recognized the limitations of mixed samples sequencing. In the present study, we firstly normalized the read count using TMM [19], and |log2(fold-change)| > 1 and *q* value < 0.005 (normalized *p* value) were used for DEGs selection, which represented accepted criteria. A total of 111 defined DEGs were identified during different stages of growth in goats. Although more biological replicates may bring more reliability to DEGs screening, the data in the present study also provides novel candidate genes for IMF deposition during growth in goats. We must also accept the limitations of RNA sequencing without technical replicates in the present study. However, we confirmed the gene expression by qPCR, which showed high correlations with RNA sequencing. As an original RNA sequencing experiment, our data were still beneficial for identifying crucial genes during muscle development in goats, despite the lack of technical replicates.

## Conclusion

This is the first study using transcriptome sequencing for expression profiling of genes during muscle development in meat goat. Based on FPKM value, out of a total of 24 689 Unigenes, 421, 305 and 447 annotated genes were found uniquely expressed in muscle tissues of kid, young and adult goats, respectively. Using series-cluster analysis, 111 DEGs were identified under 16 possible expression patterns. Co-expression analysis was used to identify node genes. Together, the results suggest candidate genes that may be important for regulation of lipid metabolism at different postnatal stages in meat goats.

## Supporting information

S1 Table Summary of genes, primers, and product sizes for quantitation real-time PCR. (DOCX)

S2 Table Top 5 differentially expressed genes between samples from kids and young goats. (DOCX)

S3 Table Top 5 differentially expressed genes between samples from kids and adult goats. (DOCX)

S4 Table Top 5 differentially expressed genes between samples from young and adult goats. (DOCX)

S5 Table Top node differentially expressed genes at different postnatal stages in goats. (DOCX)

S1 File Supporting data for all the unigenes, differentially expressed genes, and annotations. (XLSX)
