# Supplementary material for: Identification of differentially expressed genes through RNA sequencing in goats (Capra hircus) at different postnatal stages
Source: PLoS One. 2017 Aug 11;12(8):e0182602. doi: 10.1371/journal.pone.0182602 (PMC5553645; doi:10.1371/journal.pone.0182602)
Supplement: S1 Table — (DOCX) [file pone.0182602.s001.docx]

**Identification of differentially expressed genes through RNA sequencing in goats (*Capra hircus*) at different postnatal stages**

Yaqiu Lin^1¶^, Jiangjiang Zhu^1,2¶^, Yong Wang^1,2*^, Qian Li^1^ and Sen Lin^1^

^1^Key Laboratory of Sichuan Province for Qinghai-Tibetan Plateau Animal Genetic Reservation and Exploitation, Chengdu, Sichuan, P. R. China 610041

^2^Key Laboratory of State Ethnic Affairs Commission and Ministry of Education for Animal Genetics & Breeding, Chengdu, Sichuan, P. R. China 610041

* Corresponding author

E-mail: [wangyong010101@hotmail.com](mailto:wangyong010101@hotmail.com)

¶ These authors contributed equally to this work.

Funding: This work was jointly supported by the ‘Science and technology support program of Sichuan Province (2016NYZ0045)’, ‘National Natural Science Foundation of China (31672395 and 31601921)’, ‘Basic Research Programs of Sichuan Province (2016JY0147)’ and ‘Animal Science Discipline Program of Southwest University for Nationalities’ (2014XWD-S0905).

**S1 Table** Summary of genes, primers, and product sizes for quantitation real-time PCR

| Gene | Description | GenBank ID number | Primer name | Primer sequence (5´ to 3´) | Size(bp) | annealing temperature ℃ |
| --- | --- | --- | --- | --- | --- | --- |
| *C3* | complement component 3 | XM_013965236 | F. 1115 | TCATGGTGTACGTGACGAACC | 124 | 60 |
|  |  |  | R. 1238 | TGTGTGTTGATGCTTAGCTTGG |  |  |
| *CKMT2* | creatine kinase, mitochondrial 2 (sarcomeric) | XM_013965760 | F. 204 | ACAGACTATTCCCACCAAGCG | 188 | 60 |
|  |  |  | R. 391 | GCCCACGGTCTTTATGAAGG |  |  |
| *FILIP1L* | filamin A interacting protein 1 like, transcript variant X1 | XM_013972898 | F.355 | GAGGACAAGAAGCAGAGACAGC | 173 | 60 |
|  |  |  | R.527 | ACTTCATCTCGAGCCTGCAGT |  |  |
| *PPIF* | peptidylprolyl isomerase F | XM_013975638 | F. 279 | GTCCACGTTCCACAGAGTGATT | 113 | 60 |
|  |  |  | R. 391 | AGTTCTCGTCAGGAAAGCGG |  |  |
| *PPP1R27* | protein phosphatase 1, regulatory subunit 27 | XM_005694060 | F. 402 | GGCAGACATTCATCAGCGAG | 186 | 60 |
|  |  |  | R. 587 | TTGAATAGCTCCACCAAGTCCT |  |  |
| *PPTC7* | PTC7 protein phosphatase homolog (S. cerevisiae) | XM_013970669 | F. 332 | GACGGTTTGTCCCAAGTAATCC | 131 | 63 |
|  |  |  | R. 462 | TAAGCGGTGGCTGGTTCTGT |  |  |
| *PDK4* | pyruvate dehydrogenase kinase, isozyme 4 | XM_005678949 | F. 609 | CTACAATGGCACAAGGAGTCCT | 174 | 60 |
|  |  |  | R. 782 | GGCTTGGGTTTCCTGTCTGTA |  |  |
| *CSRNP1* | cysteine-serine-rich nuclear protein 1, transcript variant X2 | XM_005695556 | F. 1206 | CCTGGTGTGGATGATGATGG | 123 | 60 |
|  |  |  | R. 1328 | GTGGAAGCAGCCGAGATTG |  |  |
| *PPIB* | peptidylprolyl isomerase B | XM_005685667 | F. 427 | ACACCAACGGCTCCCAGT | 143 | 60 |
|  |  |  | R. 569 | AGGCTTGTCCCGACCATC |  |  |
