# Supplementary material for: Identification of differentially expressed genes through RNA sequencing in goats (Capra hircus) at different postnatal stages
Source: PLoS One. 2017 Aug 11;12(8):e0182602. doi: 10.1371/journal.pone.0182602 (PMC5553645; doi:10.1371/journal.pone.0182602)
Supplement: S2 Table — (DOCX) [file pone.0182602.s002.docx]

**Identification of differentially expressed genes through RNA sequencing in goats (*Capra hircus*) at different postnatal stages**

Yaqiu Lin^1¶^, Jiangjiang Zhu^1,2¶^, Yong Wang^1,2*^, Qian Li^1^ and Sen Lin^1^

^1^Key Laboratory of Sichuan Province for Qinghai-Tibetan Plateau Animal Genetic Reservation and Exploitation, Chengdu, Sichuan, P. R. China 610041

^2^Key Laboratory of State Ethnic Affairs Commission and Ministry of Education for Animal Genetics & Breeding, Chengdu, Sichuan, P. R. China 610041

* Corresponding author

E-mail: [wangyong010101@hotmail.com](mailto:wangyong010101@hotmail.com)

¶ These authors contributed equally to this work.

Funding: This work was jointly supported by the ‘Science and technology support program of Sichuan Province (2016NYZ0045)’, ‘National Natural Science Foundation of China (31672395 and 31601921)’, ‘Basic Research Programs of Sichuan Province (2016JY0147)’ and ‘Animal Science Discipline Program of Southwest University for Nationalities’ (2014XWD-S0905).

**S2 Table** Top 5 differentially expressed genes between samples from kids and young goats

|  | Gene ID | Description | Gene symbols | FPKM_G | FPKM_Y | Fold_change (Y_VS_G) |
| --- | --- | --- | --- | --- | --- | --- |
|  | 102180345 | myosin, heavy chain 13, skeletal muscle | *MYH13* | 0.076 | 6.786 | 95.339 |
|  | 102168687 | interferon-induced protein with tetratricopeptide repeats 1 | *IFIT1* | 6.569 | 24.252 | 3.929 |
|  | 102180054 | methyltransferase like 21C | *METTL21C* | 30.638 | 111.358 | 3.869 |
|  | 102171409 | myosin, light chain 6B, alkali, smooth muscle and non-muscle | *MYL6B* | 410.378 | 1338.228 | 3.470 |
|  | 102188244 | folliculin interacting protein 2 | *FNIP2* | 14.565 | 42.529 | 3.108 |
|  | 102169375 | serpin peptidase inhibitor, clade E (nexin, plasminogen activator inhibitor type 1), member 1, transcript variant X1 | *SERPINE1* | 12.957 | 2.588 | 0.213 |
|  | 102188309 | early growth response 1 | *EGR1* | 27.616 | 5.780 | 0.223 |
|  | 102174498 | estrogen-related receptor gamma, transcript variant X3 | *ESRRG* | 12.230 | 3.395 | 0.295 |
|  | 102179898 | cyclin-dependent kinase inhibitor 1A (p21, Cip1) | *CDKN1A* | 65.920 | 20.536 | 0.331 |
|  | 102170869 | butyrophilin-like 9 | *BTNL9* | 17.669 | 5.587 | 0.337 |

Note: group Y, young goats; group G, kids
