# Supplementary material for: Identification of differentially expressed genes through RNA sequencing in goats (Capra hircus) at different postnatal stages
Source: PLoS One. 2017 Aug 11;12(8):e0182602. doi: 10.1371/journal.pone.0182602 (PMC5553645; doi:10.1371/journal.pone.0182602)
Supplement: S4 Table — (DOCX) [file pone.0182602.s004.docx]

**Identification of differentially expressed genes through RNA sequencing in goats (*Capra hircus*) at different postnatal stages**

Yaqiu Lin^1¶^, Jiangjiang Zhu^1,2¶^, Yong Wang^1,2*^, Qian Li^1^ and Sen Lin^1^

^1^Key Laboratory of Sichuan Province for Qinghai-Tibetan Plateau Animal Genetic Reservation and Exploitation, Chengdu, Sichuan, P. R. China 610041

^2^Key Laboratory of State Ethnic Affairs Commission and Ministry of Education for Animal Genetics & Breeding, Chengdu, Sichuan, P. R. China 610041

* Corresponding author

E-mail: [wangyong010101@hotmail.com](mailto:wangyong010101@hotmail.com)

¶ These authors contributed equally to this work.

Funding: This work was jointly supported by the ‘Science and technology support program of Sichuan Province (2016NYZ0045)’, ‘National Natural Science Foundation of China (31672395 and 31601921)’, ‘Basic Research Programs of Sichuan Province (2016JY0147)’ and ‘Animal Science Discipline Program of Southwest University for Nationalities’ (2014XWD-S0905).

**S4** **Table** Top 5 differentially expressed genes between samples from young and adult goats

|  | Gene ID | Description | Gene symbols | FPKM_Y | FPKM_C | Fold_change (C_VS_Y) |
| --- | --- | --- | --- | --- | --- | --- |
|  | 102190429 | 40S ribosomal protein S25-like | *RPS25* | 0.409 | 34.687 | 88.279 |
|  | 102190117 | cartilage oligomeric matrix protein | *COMP* | 0.667 | 7.964 | 12.415 |
|  | 102181283 | prostate androgen-regulated mucin-like protein 1 | *PARM1* | 6.309 | 45.218 | 7.454 |
|  | 102174498 | estrogen-related receptor gamma, transcript variant X3 | *ESRRG* | 3.395 | 21.389 | 6.552 |
|  | 102187236 | growth regulation by estrogen in breast cancer 1 | *GREB1* | 1.261 | 6.189 | 5.102 |
|  | 102177715 | BOLA class I histocompatibility antigen, alpha chain BL3-7-like | *LOC102177715* | 65.371 | 0.000 | 0.024 |
|  | 102169735 | AT rich interactive domain 5B (MRF1-like), transcript variant X1 | *ARID5B* | 47.741 | 8.725 | 0.190 |
|  | 102170613 | 6-phosphofructo-2-kinase/fructose-2,6-biphosphatase 3 | *PFKFB3* | 134.776 | 26.448 | 0.204 |
|  | 102186348 | cysteine-serine-rich nuclear protein 1, transcript variant X2 | *CSRNP1* | 30.112 | 6.680 | 0.231 |
|  | 102168687 | interferon-induced protein with tetratricopeptide repeats 1 | *IFIT1* | 24.252 | 6.136 | 0.263 |

Note: group Y, young goats; group C, adult goats
