# Supplementary material for: Identification of differentially expressed genes through RNA sequencing in goats (Capra hircus) at different postnatal stages
Source: PLoS One. 2017 Aug 11;12(8):e0182602. doi: 10.1371/journal.pone.0182602 (PMC5553645; doi:10.1371/journal.pone.0182602)
Supplement: S5 Table — (DOCX) [file pone.0182602.s005.docx]

**Identification of differentially expressed genes through RNA sequencing in goats (*Capra hircus*) at different postnatal stages**

Yaqiu Lin^1¶^, Jiangjiang Zhu^1,2¶^, Yong Wang^1,2*^, Qian Li^1^ and Sen Lin^1^

^1^Key Laboratory of Sichuan Province for Qinghai-Tibetan Plateau Animal Genetic Reservation and Exploitation, Chengdu, Sichuan, P. R. China 610041

^2^Key Laboratory of State Ethnic Affairs Commission and Ministry of Education for Animal Genetics & Breeding, Chengdu, Sichuan, P. R. China 610041

* Corresponding author

E-mail: [wangyong010101@hotmail.com](mailto:wangyong010101@hotmail.com)

¶ These authors contributed equally to this work.

Funding: This work was jointly supported by the ‘Science and technology support program of Sichuan Province (2016NYZ0045)’, ‘National Natural Science Foundation of China (31672395 and 31601921)’, ‘Basic Research Programs of Sichuan Province (2016JY0147)’ and ‘Animal Science Discipline Program of Southwest University for Nationalities’ (2014XWD-S0905).

**S5 Table** Top node differentially expressed genes at different postnatal stages in goats

|  | Number | Gene ID | Number of correlated genes | Description | Gene symbols | FPKM_G | FPKM_Y | FPKM_C |
| --- | --- | --- | --- | --- | --- | --- | --- | --- |
|  | 1 | 102171870 | 54 | creatine kinase, mitochondrial 2 (sarcomeric) | *CKMT2* | 500.072 | 438.113 | 899.652 |
|  | 2 | 102190015 | 54 | pyruvate dehydrogenase kinase, isozyme 4 | *PDK4* | 1365.758 | 1498.209 | 641.702 |
|  | 3 | 102181283 | 53 | prostate androgen-regulated mucin-like protein 1 | *PARM1* | 12.660 | 6.309 | 45.218 |
|  | 4 | 102186348 | 53 | cysteine-serine-rich nuclear protein 1, transcript variant X2 | *CSRNP1* | 32.734 | 30.112 | 6.680 |
|  | 5 | 102190117 | 53 | cartilage oligomeric matrix protein | *COMP* | 0.600 | 0.667 | 7.964 |
|  | 6 | 102190429 | 53 | 40S ribosomal protein S25-like | *RPS25* | 0.257 | 0.409 | 34.687 |
|  | 7 | 102168299 | 52 | regulator of calcineurin 1 | *RCAN1* | 170.308 | 157.919 | 57.327 |
|  | 8 | 102170177 | 52 | protein phosphatase 1, regulatory subunit 27 | *PPP1R27* | 736.973 | 811.742 | 1628.235 |
|  | 9 | 102176494 | 52 | jumonji domain containing 1C, transcript variant X1 | *JMJD1C* | 14.637 | 17.145 | 7.536 |
|  | 10 | 102176560 | 52 | filamin A interacting protein 1-like, transcript variant X1 | *FILIP1L* | 47.148 | 52.401 | 23.345 |
|  | 11 | 102177922 | 52 | clustered mitochondria (cluA/CLU1) homolog | *CLUH* | 35.098 | 28.229 | 67.259 |
|  | 12 | 102178281 | 52 | solute carrier family 20 (phosphate transporter), member 1 | *SLC20A1* | 21.389 | 25.463 | 9.810 |
|  | 13 | 102180788 | 52 | PTC7 protein phosphatase homolog (S. cerevisiae) | *PPTC7* | 41.316 | 30.679 | 70.059 |
|  | 14 | 102187374 | 52 | peptidylprolyl isomerase F | *PPIF* | 13.933 | 8.630 | 31.344 |
|  | 15 | 102188033 | 52 | complement component 3 | *C3* | 20.161 | 20.158 | 8.998 |
|  | 16 | 102188626 | 52 | sperm antigen with calponin homology and coiled-coil domains 1 | *LOC102188626* | 17.881 | 20.587 | 58.185 |

Note: group G, kid goats; group Y, young goats; group C, adult goats
